# Economic Diversification Supported the Growth of Mongolia’s Nomadic Empires

**DOI:** 10.1038/s41598-020-60194-0

**Published:** 2020-03-03

**Authors:** Shevan Wilkin, Alicia Ventresca Miller, Bryan K. Miller, Robert N. Spengler, William T. T. Taylor, Ricardo Fernandes, Richard W. Hagan, Madeleine Bleasdale, Jana Zech, S. Ulziibayar, Erdene Myagmar, Nicole Boivin, Patrick Roberts

**Affiliations:** 1 Max Planck Institute for the Science of Human History, Department of Archaeology, Jena, Germany; 2 University of Michigan, Department of Anthropology, Ann Arbor, Michigan USA; 3 University of Colorado, Department of Anthropology, Museum of Natural History, Boulder, CO USA; 4 School of Archaeology, University of Oxford, Oxford, UK; 5 Faculty of Arts, Masaryk University, Brno, Czech Republic; 6 Max Planck Institute for the Science of Human History, Department of Archaeogenetics, Jena, Germany; 7 Institute of Archaeology and Ethnology, Mongolian Academy of Sciences, Jukoviin orgon chuloo 77, Ulaanbaatar, Mongolia; 8 National University of Mongolia, Ulaanbaatar, Mongolia; 9 School of Social Science, The University of Queensland, Brisbane, Australia; 10 Department of Anthropology and Archaeology, University of Calgary, Calgary, Alberta Canada; 11 Department of Anthropology, National Museum of Natural History, Smithsonian Institution, Washington, D.C., USA

**Keywords:** Population dynamics, Stable isotope analysis, Archaeology

## Abstract

Populations in Mongolia from the late second millennium B.C.E. through the Mongol Empire are traditionally assumed, by archaeologists and historians, to have maintained a highly specialized horse-facilitated form of mobile pastoralism. Until recently, a dearth of direct evidence for prehistoric human diet and subsistence economies in Mongolia has rendered systematic testing of this view impossible. Here, we present stable carbon and nitrogen isotope measurements of human bone collagen, and stable carbon isotope analysis of human enamel bioapatite, from 137 well-dated ancient Mongolian individuals spanning the period c. 4400 B.C.E. to 1300 C.E. Our results demonstrate an increase in consumption of C_4_ plants beginning at c. 800 B.C.E., almost certainly indicative of millet consumption, an interpretation supported by archaeological evidence. The escalating scale of millet consumption on the eastern Eurasian steppe over time, and an expansion of isotopic niche widths, indicate that historic Mongolian empires were supported by a diversification of economic strategies rather than uniform, specialized pastoralism.

## Introduction

Mongolian empires, such as the Xiongnu and Mongols, are some of the most renowned imperial entities in public and academic thought. This is, in part, due to their historical portrayal as highly mobile, predatory horseback polities with a specialized dairy and meat-based economy^1–4^, an image that is perpetuated in cinema, novels, and documentaries alike. While such stereotypes likely arose from the hyperbolized accounts of neighboring adversaries, starting with the Han, who fought against the Xiongnu^5^, they have persisted and now pervade academic evaluations of the economic basis of these ancient peoples. The modern economic focus on pastoralism in rural areas of Mongolia today is frequently viewed as a relic of the past and has been drawn upon to interpret the often-fragmentary archaeological record of this region^6,7^, although ethnoarchaeological approaches often ignore the role of urban markets and motorized transport in modern mobile pastoralism. The view of uniformly specialized pastoral economies has also furthered the scholarly fascination with historical Mongolian populations, resurrecting the long-standing question of whether an empire can meet the costs and challenges of long-term political and economic organization in the absence of grain surpluses^6,7^.

Empires are, however, inherently complex and, by definition, extend their control over multiple societies, cultures, and economies, as well as heterogeneous landscapes^8–10^. Crop surplus has traditionally been viewed as an essential component of stable political entities and complex imperial food production and procurement systems are often over-simplified by historians and archaeologists, leading to their characterization as single-resource systems (such as maize for the pre-Columbian empires of South America). Refined analyses generally reveal diverse and dynamic economies supporting imperial expansions, which draw together a variety of food sources^11^. As a consequence, it is perhaps unsurprising that archaeological, archaeobotanical, and historical records are beginning to strongly hint at the possibility that historical Mongolian empires were not solely reliant on dairy pastoralism, but also featured agriculture, as well as craft specialization, and participation in trade systems that spanned thousands of kilometers^12–14^.

Of particular interest in this context has been the growing archaeobotanical evidence from across Central Asia that demonstrates an influx of millet, both broomcorn (*Panicum miliaceum* L.) and foxtail (*Setaria italica* L.), and other domesticated grains in the surrounding steppe lands of Siberia, Kazakhstan, and northwestern China during the second and first millennia B.C.E.^15–24^. There are some archaeobotanical data suggesting the use of crops in Mongolia starting around *c*. 100 B.C.E. – 200 CE^25,26^, though these have been dismissed as reflective of trade rather than local production^6,27^. Overall, due to issues of wind deflation and a lack of sampling during excavation, archaeobotanical evidence from Mongolia is severely lacking. Moreover, where present, it is difficult to determine the degree to which an archaeobotanical assemblage represents overall dietary reliance. To date, there have been no systematic, direct analyses of the consumption of domesticated crops among peoples in this region over the past three millennia, leaving the economic basis for some of the world’s most famous empires unresolved.

Stable isotope analysis of archaeological human and associated faunal remains has emerged as an increasingly powerful methodology for tracking palaeodietary and subsistence change in Central and East Asia^23,28–31^. The distinction in stable carbon isotope ratios (δ^13^C) between C_3_ plants on the one hand – including crops such as rice (*Oryza sativa* L.), wheat (*Triticum* spp. L.) and barley (*Hordeum vulgare* L.) – and C_4_ plants on the other – including millets and maize (*Zea mays* L.) – can be tracked through the bone collagen and bioapatite of humans relying on these resources and/or the animals feeding off of them^32^. While bone collagen δ^13^C is primarily derived from the protein portion of the diet, tooth enamel bioapatite δ^13^C reflects the whole diet^33^. Stable nitrogen isotope (δ^15^N) analysis provides additional insights into the trophic level position of an individual, orienting them within the local food chain^34^.

It is our aim to discover when Eastern Steppe populations began utilizing cultivated C_4_ resources (i.e. millet and millet-based foods). We are especially interested in dietary trends during the Xiongnu and Mongol imperial periods, as there has long been a dominant assumption that these empires wholly depended on dairy pastoralism. We use stable carbon isotope analysis of human tissues to directly test whether, in line with some previously published archaeobotanical and historic evidence, the Xiongnu and Mongol empires in fact relied quite significantly on millet-based agricultural systems. We present δ^13^C and δ^15^N analysis of human bone collagen and δ^13^C and δ^18^O analysis of human tooth enamel bioapatite from 137 previously-excavated individuals from across Mongolia dated to between c. 4400 B.C.E. and 1375 C.E. in order to directly assess changing diets through the region’s key imperial transitions.

## Results

### Preservation of samples

**Figure 1:**
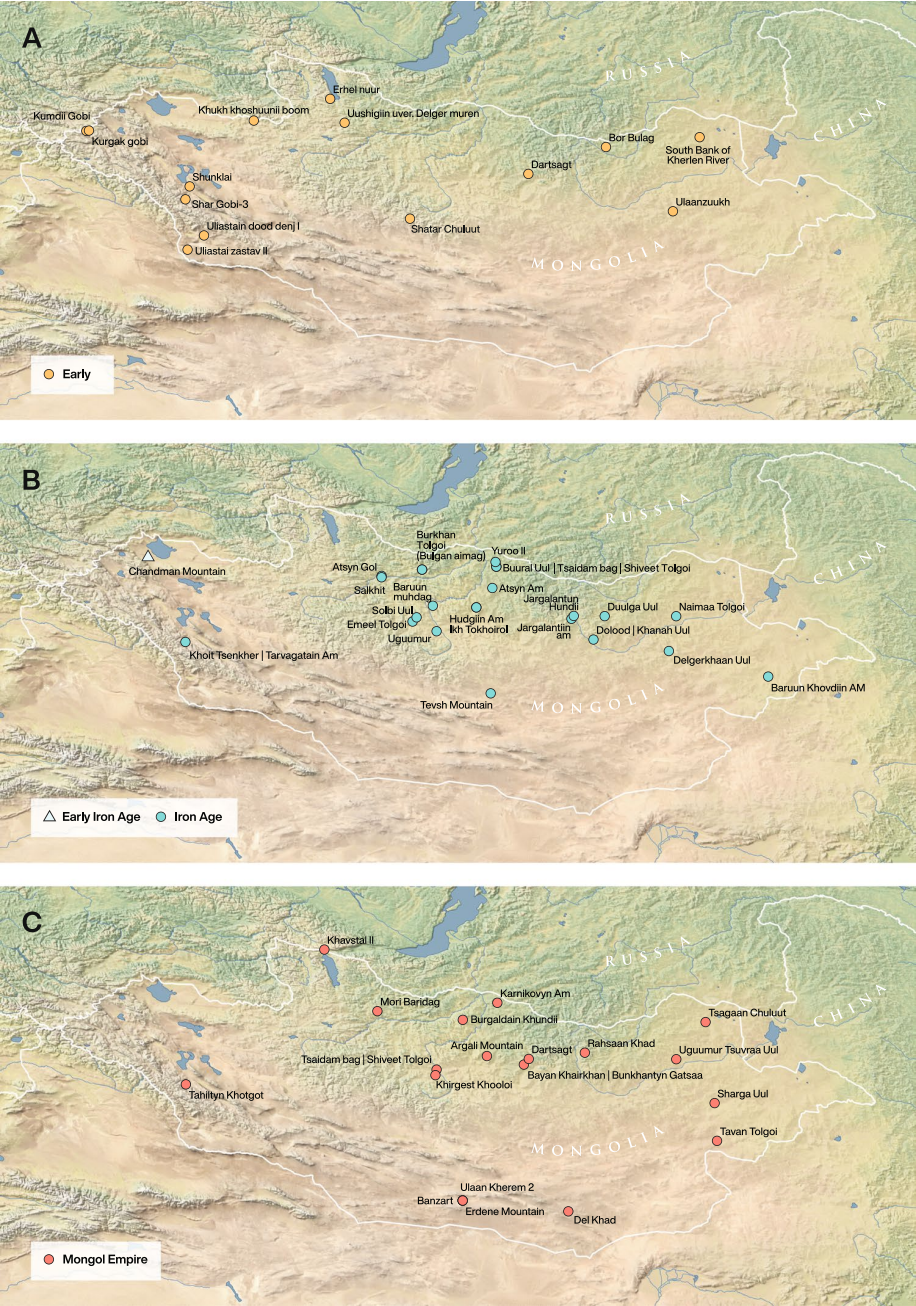
Maps of sites used in this study. These maps were created for this study and were produced using QGIS 3.0^89^ https://qgis.org/en/site and using the Natural Early Data maps from https://www.naturalearthdta.com/downloads/ by Shevan Wilkin and Michelle O’Reilly (Graphic Designer for the Max Planck Institute for the Science of Human History, Jena, Germany).

We analyzed 80 bone collagen and 108 dental enamel samples from 137 individuals from 60 archaeological sites (Tables 1–4; Fig. 1). Samples were separated into four chronological periods based on relative and absolute dating (Early [Neolithic - Bronze Age], Early Iron, Xiongnu, and Mongol; see Supplementary Table S4 for AMS dates). As there is only a single individual from the Neolithic period (c. 4400–3000 B.C.E.), this sample was combined with Bronze Age individuals dating to prior to 800 B.C.E. (n = 23; collagen n = 14, enamel n = 16) to create a single period labelled as ‘Early’. The Iron Age samples were split into two chronological periods, corresponding to the pre-imperial Early Iron Age (*c*. 800–200 B.C.E.) and the Xiongnu (*c*. 200 B.C.E. – 250 C.E.). The Early Iron Age samples include 16 individuals (collagen n = 7, enamel n = 16) from one site. From the subsequent Xiongnu, we analyzed 59 individuals (collagen n = 23, enamel n = 54) from 28 sites. Individuals from the later Mongol Empire (*c*. 1200–1375 C.E.) are grouped together and consist of 28 individuals (collagen n = 28, enamel n = 21) from 19 sites.

**Table 1:** Average bone collagen values for individuals in this study by time period, individual values presented in Supplementary Table 1 (results include additional individuals from previously published articles^20,29,30,44^).

Time Period	Date Range	Mean δ13C (‰) (VPDB); SD	δ13C (‰) (VPDB) Range	Mean δ15N (‰) (AIR); SD	δ15N (‰) (AIR) Range
Early (n = 14)	4400–800 B.C.E.	−17.3 ± 0.8	−18.5 – 16.2	+12.8 ± 1.0	+11.0 – +14.6
Early Iron (n = 7)	800–200 B.C.E.	−16.0 ± 0.8	−16.8 – 14.8	+13.6 ± 1.1	+12.2 – +14.8
Xiongnu (n = 47)	200 B.C.E-250 C.E.	−16.0 ± 1.3	−18.5 – 13.1	+13.2 ± 1.3	+7.9 – +15.5
Mongol (n = 38)	1200–1375 C.E.	−16.5 ± 1.7	−20.4 – 12.4	+12.8 ± 1.7	+6.9 – +16.2
Faunal* (n = 53)	2000 B.C.E. – 200 C.E.	−18.4 ± 1.8	−21.8 – 13.16	+8.1 ± 2.4	+3.5 – +12.6

**Table 2:** Average tooth enamel bioapatite values by time period.

Time Period	Date Range	δ^13^C (‰) (VPDB)	δ^13^C (‰) (VPDB) range	δ^18^O (‰) (VPDB)	δ^18^O (‰) (VPDB) range
Early (n = 17)	4400–800 BC	−12.9 ± 0.8	−14.3 – −11.9	−10.6 ± 1.0	−11.9 – −8.1
Early Iron (n = 14)	800–200 BC	−11.0 ± 2.1	−13.3 – −5.7	−11.0 ± 0.9	−12.1 – −9.5
Xiongnu (n = 56)	200 BC-250 AD	−11.2 ± 2.3	−14.9 – −3.1	−10.5 ± 1.7	−15.2 – −7.2
Mongol (n = 21)	1200–1375 AD	−11.3 ± 1.9	−15.1 – −6.8	−10.8 ± 2.0	−13.6 – −6.5

**Table 3:** Average human and faunal bone collagen δ^13^C and δ^15^N values between the steppe (>250 mL annual precipitation) and dry (<250 mL annual precipitation) regions.

Time Period	Steppe	Mean δ^13^C (‰) (VPDB); SD	Mean δ^15^N (‰) (AIR); SD	Dry	Mean δ^13^C (‰) (VPDB); SD	Mean δ^15^N (‰) (AIR); SD
Early	Steppe (n = 2)	−18.2 ± 0.1	+11.6 ± 0.8	Dry (n = 11)	−17.2 ± 0.7	+13.1 ± 0.8
Early Iron	Steppe (n = 6)	−16.3 ± 0.7	+13.9 ± 1.0	Dry (n = 8)	−16.2 ± 0.9	+13.5 ± 1.1
Xiongnu	Steppe (n = 17)	−16.1 ± 1.2	+12.2 ± 1.1	Dry (n = 30)	−16.1 ± 1.1	+13.7 ± 0.8
Mongol	Steppe (n = 10)	−16.8 ± 2.2	+11.6 ± 2.0	Dry (n = 25)	−16.1 ± 1.4	+13.2 ± 1.3
Faunal	Steppe (n = 34)	−19.3 ± 1.3	+6.8 ± 1.9	Dry (n = 19)	−17.2 ± 1.5	+9.1 ± 2.3

**Table 4:** Average human dental enamel δ^13^C and δ^18^O values between the steppe (>250 mL annual precipitation) and dry (<250 mL annual precipitation) regions.

Time Period	Steppe	Mean δ^13^C (‰) (VPDB)	δ^18^O (‰) Average	Dry	Mean δ^13^C (‰) (VPDB)	δ^18^O (‰) Average
Early	Steppe (n = 3)	−14.1 ± 0.2	−11.1 ± 0.3	Dry (n = 4)	−13.0 ± 0.5	−10.4 ± 1.6
Early Iron	Steppe (n = 15)	−11.1 ± 2.0	−10.1 ± 3.6	Dry (n = 0)	n.d.	n.d.
Xiongnu	Steppe (n = 29)	−10.7 ± 2.2	−10.6 ± 1.5	Dry (n = 4)	−12.3 ± 1.0	−10.5 ± 1.5
Mongol	Steppe (n = 5)	−10.6 ± 2.3	−12.6 ± 1.0	Dry (n = 5)	−11.7 ± 1.1	−9.6 ± 2.6

All of the human bone collagen samples included in this study had atomic C:N ratios between 3.1 and 3.5 and were thus within the accepted range for good collagen preservation^35^ (Supplementary Table S1). The collagen yields of these samples ranged between 6 and 30%, with none falling below 1%, a further check of data quality^35^. Furthermore, the majority of collagen samples have greater than 11% N and greater than 30% C, within acceptable ranges^36^. Each bone collagen sample was run in duplicate, and averages are presented in Supplementary Table S1 along with their standard deviation.

### Bone collagen carbon and nitrogen stable isotope results

δ^13^C and δ^15^N results from human bone collagen are grouped into four chronological periods, as detailed above, for comparative analysis. The pre-Bronze and Bronze Age average values are the closest to those of the average faunal values, although the faunal values have a higher standard deviation. The widest range of carbon and nitrogen isotope values were found in the Xiongnu and Mongol Period populations. For all of the individual δ^13^C and δ^15^N values from each time period see Supplementary Table 1.

### Dental enamel carbon stable isotope results

The data from δ^13^C values of human enamel bioapatite are divided into the same chronological periods as the bone collagen data. The Xiongnu population had the largest range of stable carbon isotope values, followed by the Mongol period and Early Iron Age (Table 2). The pre-Bronze/Bronze period had the lowest range of stable carbon isotope values when compared to the later populations. For all of the individual δ^13^C and δ^18^O values from each time period see Supplementary Table 2 (Samples with both collagen and enamel Supplementary Table 3).

### Environmental differences

As stable carbon and nitrogen isotope values may vary in different environments (i.e. temperature and aridity), to adequately assess human δ^13^C and δ^15^N values from normal steppe (>200 mL of annual precipitation) and dry (<200 mL of annual precipitation) regions, we also determined the average values for each environmental type (Table 3). In these tables we have separated the previously published faunal stable carbon and nitrogen isotope values into the “steppe” or “dry” regions according to modern annual rainfall^37,38^.

### Statistical tests

**Figure 2:**
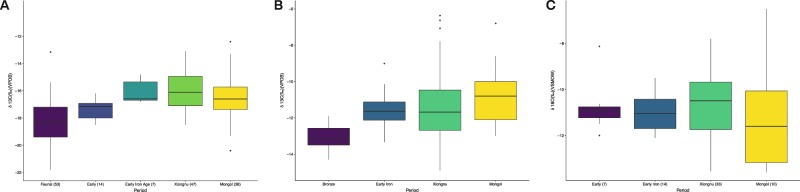
Boxplots showing the range of carbon values for all individuals from each period. Outliers are shown as individual data points. (**A**) Comparison of the bone collagen carbon values for humans and fauna. Faunal data derives from previously published data^20,29,30,44^, and all human data is from this study. (**B**) Difference in human enamel values between Early, Early Iron, Xiongnu, and Mongol periods. The Early Iron, Xiongnu, and Mongol period average values are significantly higher than the Early period average. (**C**) Boxplots showing the range of oxygen values from enamel samples. There are no significant differences between any of the time periods.

Boxplots of our results can be found in Fig. 2A–C, and statistical comparisons between the time periods can be found in Supplementary Table 5 (δ^13^C bone collagen), Supplementary Table 6 (δ^13^C enamel bioapatite), and Supplementary Table 7 (δ^15^N bone collagen). For the bone collagen data, both the Xiongnu and the Mongol average δ^13^C values were significantly higher than those of Early individuals (p < 0.05). The same trend was seen for tooth enamel δ^13^C, with Early Iron, Xiongnu, and Mongol samples having δ^13^C significantly higher than that of the Early group (for the overall p < 0.05, and the specific pairwise comparisons are available in Supplementary Table 6). There was no significant difference between average dental enamel values for the Early Iron, Xiongnu, and Mongol periods δ^13^C (p > 0.05). Bronze Age δ^15^N values were also significantly higher (p < 0.05) than those of the Early Iron, Xiongnu, and Mongol periods (Supplementary Table 7).

### Isotopic temporal trends in Mongolia and environmental impacts

Higher δ^13^C values in individuals from the Early Iron Age, Xiongnu, and Mongol periods could be the product of the increased direct consumption of C_4_ crops or wild plants or animals consuming C_4_ plants. It should also be understood that both Mongolians and foreign travellers would have been moving within and outside of the imperial borders, and dietary intake likely varied greatly in different regions. In areas with environmentally-linked variation in wild C_4_ and C_3_ plant distributions, such as Mongolia, it is important to rule out a climatically driven change (see Supporting Information Text 1). Modern plant samples from Mongolia have yielded δ^13^C values ranging from −28.3 to −23.4‰ for C_3_ photosynthetic pathways and an average δ^13^C of −14.7‰ for plants following the C_4_ photosynthetic pathway^39^. Notably, wild C_4_ plants make up a much smaller proportion of Mongolian and other Central Asian environments than C_3_ plants^40,41^. Overall, contemporary studies suggest that leaf δ^13^C values decrease with increasing mean annual precipitation^42^, both as a product of reduced C_4_ plants in wetter landscapes and aridity-driven changes in δ^13^C among C_3_ plants (see Supplemental Text S1).

While C_4_ plants make up a relatively limited portion of the biotic community today, we established local isotopic baselines for Mongolia in the past using archaeological fauna in order to determine if shifts in δ^13^C values through time are the product of environmental variations or social and economic choices. Isotopic studies of modern and archaeological herd animals have shown differences in δ^13^C values between more and less arid regions^43–45^, and that there is variation in the availability of C_3_ and C_4_ plants across the country^46,47^. While there were no fauna associated with the human remains collected for this study, we were able to use previously published faunal stable isotope data from the Minusinsk Basin of Siberia (just north of Mongolia)(MNSK, AD, AM; n = 21)^20,29^, the Gobi (BGC; n = 14)^30,48^, Gobi-Altai (SBR; n = 5)^30^, and north central Mongolia (EG; n = 13)^30^ areas to show that regional herbivores generally consumed C_3_ plants, with some having higher stable carbon isotope values, indicative of C_4_ plant consumption, in the hyper-arid desert regions^30,48^.

**Figure 3:**
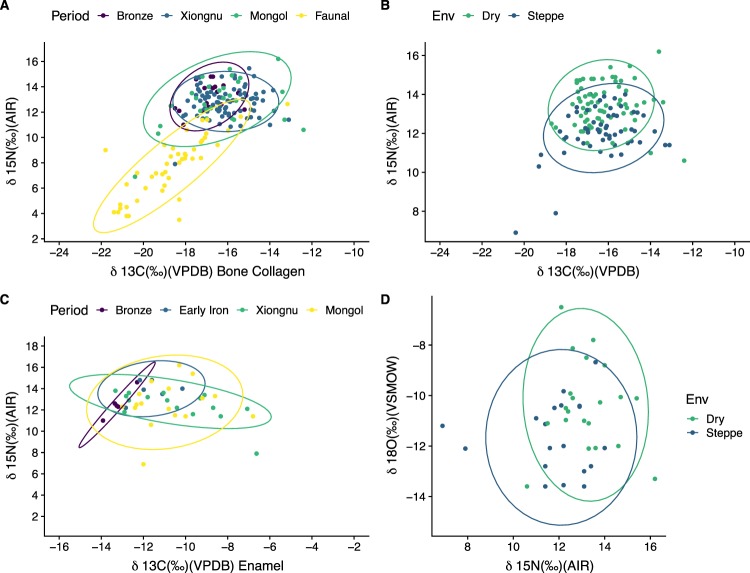
Carbon and nitrogen values from bone collagen with ellipses showing ranges at 95% confidence. (**A**) Individuals included in this study as well as humans and faunal values from previously published data^31^ (**B**) Humans included in this study showing the variation between those in the “Dry” and “Steppe” zones. “Dry” sites have less than 250 mm of annual precipitation, “Steppe” sites have over 250 mm of precipitation per year. (**C**) δ^15^N from bone collagen versus δ^13^C values from dental enamel demonstrating the shift from primarily C_3_ reliant diets in the Early period to a wider range of carbon and nitrogen values, indicating an increase in the diversity of diets in the later three periods. (**D**) δ^18^O from dental enamel versus δ^15^N from bone collagen showing the values in “Dry” and “Steppe” areas.

Statistical tests further support this assessment, with humans having higher δ^13^C values than the available fauna in all periods, with the greatest difference occurring in the Xiongnu and Mongol periods (p ≤ 0.005)(Figs. 2A and 3A). For terrestrial faunal remains (Fig. 3A), there is a significant correlation between δ^15^N and δ^13^C bone collagen values (R^2^ = 0.64, p-value <0.001) which is a product of higher levels of aridity leading to a higher availability of C_4_ plants in the natural vegetation cover. However, no such correlation is observed in humans (Fig. 3A,B), either between δ^15^N and δ^13^C bone collagen values (R^2^ = 0.01, p-value = 0.15) or between δ^15^N bone collagen and δ^13^C enamel values (R^2^ = 0.05, p-value = 0.13). Given this, alongside the consistent elevation of human δ^13^C values over the available fauna δ^13^C values, this indicates that higher δ^13^C values in human bone collagen and enamel is a product of direct consumption of non-wild C_4_ plants.

**Figure 4:**
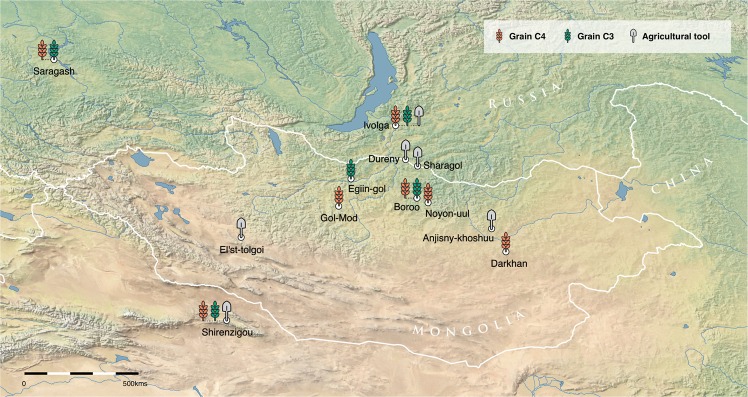
Sites in and around Mongolia with archaeological or archaeobotanical evidence for C_3_ (wheat and barley) and C_4_ (broomcorn and foxtail millet) grain cultivation during the Iron Age. This map was newly created for this study and produced using QGIS 3.0^89^ https://qgis.org/en/site and using the Natural Early Data maps from https://www.naturalearthdta.com/downloads/ by Shevan Wilkin, Bryan K. Miller, and Michelle O’Reilly (Graphic Designer for the Max Planck Institute for the Science of Human History, Jena, Germany).

Mean bone collagen δ^13^C values for faunal remains from steppe regions are typically C_3_ (−19.3 ± 1.3‰), and the stable carbon isotopic offset between bone collagen of herbivores and carnivores is *c*. 1‰^49^. Thus, human bone collagen steppe samples dating to the Early period (prior to 800 B.C.E.) do not show δ^13^C values indicative of a millet dietary contribution (−18.3 ± 0.1‰). This same offset applies to human bone collagen samples from dry regions from all periods since these are elevated by up to 1‰ (Early −17.2 ± 0.7‰; Early Iron = −16.2 ± 0.9‰; Xiongnu −16.1 ± 1.1‰; Mongol −16.1 ± 1.4‰) when compared to the bone collagen mean for faunal samples from dry regions (−17.2 ± 1.5‰). However, for later time periods in steppe regions average human bone collagen δ^13^C values are elevated by c. 3‰ (Early Iron −16.3 ± 0.7‰; Xiongnu −16.1 ± 1.2‰; Mongol −16.8 ± 2.2‰) when compared to faunal values. Thus, the higher (*c*. 2‰) human bone collagen δ^13^C values observed for later periods in steppe regions when compared to the early period is indicative of a temporal increase in millet-based food consumption. Furthermore, given that bone collagen reflects primarily consumed protein, and that millet has a poor protein content, the dietary caloric contribution from millet was likely much  higher than its protein contribution^50^. This is corroborated by human enamel δ^13^C values given that this isotopic proxy reflects the carbon dietary mix^50^. Steppe human enamel samples for the later periods show mean δ^13^C values higher by *c*. 3.5‰ when compared to the Early period. For dry areas, mean human enamel samples δ^13^C values are higher by *c*. 1‰ when compared to the Early period, which indicates a temporal increase in millet-based food consumption although considerably smaller than that observed in the steppe regions as shown also in the model estimates for millet caloric contributions (Fig. 4).

Further evidence for C_4_ plant consumption is offered by the distribution of isotopic values. For faunal remains there is a positive significant correlation (R^2^ = 0.67, p-value <0.05, correlation coefficient =1.126) between δ^15^N and δ^13^C bone collagen values which is expected given that an increase in aridity leads to a higher availability of C_4_ plants in the vegetation cover. A similar correlation, albeit with isotopic offsets, would be expected if humans relied exclusively on animal products. However, no clear environmentally-driven correlation is observed for the human groups. There is no significant correlation for the Early (R^2^ = 0.65, p-value <0.08, correlation coefficient = 0.65) and Xiongnu (R^2^ = 0.0, p-value <0.46, correlation coefficient = 0.11) periods, and although the correlation is significant for the Mongol period (R^2^ = 0.13, p-value <0.02, correlation coefficient = 0.39), it only explains 39% of the variability. For similar δ^15^N bone collagen values across the human individuals, there are wide ranges in δ^13^C collagen values. Whereas during the Xiongnu period one can observe a significant negative correlation (R^2^ = 0.0, p-value <0.46, correlation coefficient = 0.11), which implies the contribution from a food source with higher δ^13^C values but lower δ^15^N values when compared to animal food sources. These isotopic relationships are indicative of varying individual intake of a food with elevated δ^13^C values, such as millet, and having relatively uniform δ^15^N and δ^13^C values across regions with varying levels of aridity.

### Bayesian spatial modelling of C_4_ plant caloric consumption

**Figure 5:**
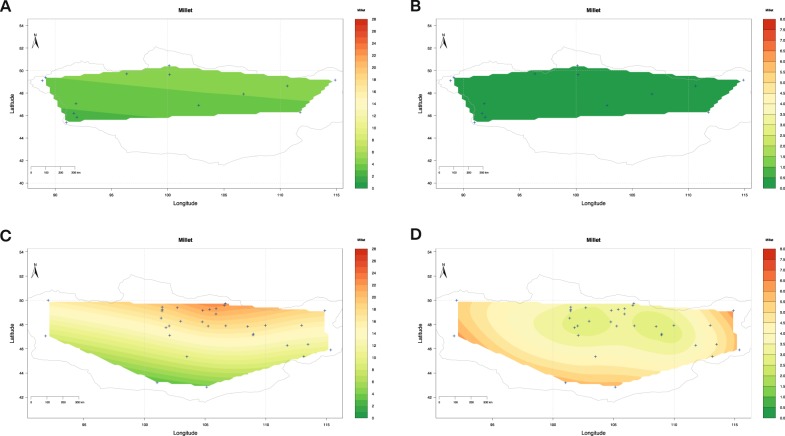
Dietscape representing average millet caloric consumption per capita. (**A**) Average millet caloric consumption for the Early period estimated through Bayesian modelling of dental bioapatite carbon stable isotope values. (**B**) Standard error of the mean for millet caloric consumption average during the Early period. (**C**) Average millet caloric consumption for the Late period estimated through Bayesian modelling of dental bioapatite carbon stable isotope values. (**D**) Standard error of the mean for millet caloric consumption average during the Late period.

To further confirm that the increased δ^13^C values in human bone collagen and tooth enamel through time is a product of the consumption of crops rather than changing availabilities of baseline C_4_/C_3_ plant ratios or the availability of samples in different local environments, we developed a Bayesian model to produce a C_4_ dietscape, representing estimates of spatial distribution of C_4_ plants based on per capita caloric consumption (See SI for detailed discussion). Stable carbon isotope data of dental enamel was used, and individuals were separated into two periods, Early (Neolithic - Bronze Age) or Late (Xiongnu, and Mongol). The results for the two models show that during the Early period C_4_ caloric contributions were very low across Mongolia, likely including consumption of local plants and livestock consuming natural vegetation, with mean estimates varying between *c*. 2.5 and 5.0% of calories (interpolation 1-sigma uncertainty up to 0.5% calories) (Fig. 5A,B). During the later periods, the variability in millet-based food consumption increases considerably as shown by the range in the mean estimate (between 3 and 26% of per capita millet calories) and in the 1-sigma interpolation uncertainty for each location (between *c*. 3 and 6% of per capita millet calories) (Fig. 5C,D). The C_4_ plant dietscape for the late period also shows that millet consumption is concentrated in central northern Mongolia (reaching the highest mean value [26% per capita millet calories]), an area where environmental increase of carbon values would not be expected naturally (Figs. 4 and 5).

## Discussion

### Isotopic indicators of diet through time in mongolia

Our results clearly demonstrate an increase of human consumption of C_4_ plants during the imperial periods in ancient and historic Mongolia (Figs. 2, 3, and 5). While high δ^15^N values in human bone collagen relative to the faunal data (Fig. 2A and 3A) supports evidence for human reliance on dairy and meat products throughout the periods under study, the change in C_4_ plant consumption represents the major dietary shift within this timeframe. The significant decrease in δ^15^N values in the later periods, in comparison to the Bronze Age, further supports this point, potentially indicating reduced consumption of meat and milk and increased consumption of grains. Moreover, comparisons with faunal datasets and environmental background data allows us to confidently state that this shift is a consequence of increasing rates of consumption of C_4_ resources. Stable carbon isotope values from individuals before the Early Iron Age show little indication of C_4_ plant consumption beyond the local natural baseline identified through the fauna from similar environments. Individuals from the cemetery of Chandman Mountain (c. 900–400 B.C.E.) analyzed in this study show the first visible evidence of C_4_ plant consumption as part of a mixed agro-pastoral diet. However, this particular site in northwest Mongolia is more a part of the Minusinsk Basin region of southern Siberia, an area where millet consumption was common by the Late Bronze Age, than the rest of Mongolia. Previously published contemporaneous human and faunal isotope analyses within this region are indicative of animals consuming primarily C_3_ plants^20^, with the human population postulated as having a diet additionally composed of C_4_ plants in concert with meat and milk.

In the rest of Mongolia during the tenure of the Xiongnu and Mongol empires human stable carbon values became more varied with increasing numbers of individuals displaying bone collagen and tooth enamel δ^13^C values suggestive of moderate to high C_4_ plant consumption, with the number of individuals with such values reaching their peak during these imperial periods. We also observe the largest range and diversity of δ^13^C and δ^15^N values during the imperial periods. This is likely due to diverse subsistence strategies being pursued across each empire, reflecting different environmental zones and levels of imperial support. This is result of the extensive range of each empire, and includes the knowledge that not everyone that died in Mongolia would have been “Mongolian”, but these individuals likely lived and died within the empires. Since the majority of the individuals analyzed were excavated from elite imperial tombs, and human remains representing other sectors of society are lacking at present, attributing all outliers to non-local outsiders would be to dismiss the agency of Mongolian populations and provide something of a ‘colonial’ narrative.

Individual bone collagen and tooth enamel δ^13^C values for the Xiongnu and Mongol empires range between those indicative of a pure C_3_ diet to those that suggest heavy C_4_ plant consumption. Interestingly, during this period, a few individuals had δ^13^C values lower than those of the Early period which, alongside lower δ^15^N values, indicates a staple intake of C_3_ plants, likely crops such as wheat and barley. Historical and archaeobotanical sources suggest that cereal crops were commonly cultivated or obtained through trade during the Mongol period^13,51–56^. In addition to grains, carbonized fruit and nut remains have been recovered from sediments at the Mongol capital of Kharakhorum (also used during the Mongol rule in the Yuan Dynasty) showing the diversity of imported plants through the presence of rice (*Oryza sativa* L.), over a dozen cultivated fruits, including grapes (*Vitis vinifera* L.), figs (*Ficus carica* L.), and jujube (*Ziziphus jujube* Mill.), as well as vegetable and oil-seed crops. There are also remains of spices – notably a few, such as black pepper (*Piper nigrum* L.) and caraway (*Carum carvi I*.), that were imported along the trade routes with South Asia, and would have involved transport across distances of up to 2000 kilometers^12^.

The resulting bone collagen δ^13^C and δ^15^N values have been plotted to show this increase of dietary diversity over time (Fig. 2A). From our data, alongside the growing corpus of biomolecular, archaeological, and historical data, it is evident that the Xiongnu and Mongol Empires had complex imperial structures that facilitated increasingly diverse subsistence economies. The combination of crop cultivation in tandem with dairy pastoralism would have allowed these empires to sustain a diverse economic surplus that defended against livestock depletion from harsh winters, crop loss, or volatile political episodes. Diverse dietary values likely also reflect an increasingly cosmopolitan society in which dietary heterogeneity within populations increased with growing migration, trade and interaction, and the emergence of increasingly elaborated elite statuses. The diversity could also reflect temporal political shifts within the time-span covered by our sampling groups, with trade routes to Karakorum decreasing in volume during the Mongol Period after the switch of the capital in 1260 and ending with the end of the Yuan Dynasty in 1368^12,57^, for example.

### Mongolian empires in context

Historical and ethnographic research indicates the importance of pastoralism on the historic and proto-historic eastern Eurasian Steppe. Recent proteomic research has demonstrated the clear importance of dairy-based pastoralism to Mongolian dietary practices from at least c. 1500 B.C.E.^58^. Nevertheless, our data clearly highlight that pastoral lifestyles did not preclude the inclusion, and later intensification, of crop use. Millet’s suitability to arid environments combined with its short growing period is compatible with the often peripatetic, mobile lifestyles of pastoralists^22,26^. Indeed, during the Xiongnu and Mongol empires, we see clear evidence for human dietary reliance on millet in a significant proportion of individuals. Although some scholars contend that all grains were either extorted or imported from China and other exterior polities^6^, we argue that our data, alongside existing archaeobotanical and archaeological findings^25,59,60^, provide clear evidence for imperial reliance on locally grown crops in the Xiongnu and Mongol heartlands, as well as the coordination of diverse economic connections and exchanges^12^. These discoveries bolster the notion of an economically diverse population across much of Mongolian history^14,61,62^.

Agricultural tools for plowing, hoeing, and grinding have been uncovered from permanent Xiongnu settlements in Mongolia, implying local plant cultivation and processing^63^, and charred remains of millet, barley, and wheat grains have been recovered through flotation at pit-house villages at Boroo^64^ and ephemeral campsites^61^. Studies in the Egiin Gol valley and at the large site complex of Ivolga have illustrated the presence of long-season cereal crops (wheat and barley) in the Iron Age, which represent more labor investment in farming practices than millets^61,63^. At Ivolga, this occurs alongside evidence of ploughshares at permanent settlements^63^, as well as written accounts of crops suitable for the northern steppe being managed by imperial Xiongnu administrators, such as the ‘Lord of Millet Distribution’, referred to in the 1st century C.E. Chinese accounts^54^. Millet grains, still articulated in their chaff, have been found within the graves of Xiongnu rulers at Gol Mod and Noyon Uul^27^ as well as of local elites throughout the steppe^60^. There are also uncharred grains found within Xiongnu pit-house villages, all of which were unprocessed (i.e. with palea and lemma) and thus most likely not transported long distances^60^ instead representing local production and consumption (Fig. 4).

Scholars working in Mongolia have extensively discussed the formation of hierarchical political systems and greater concentrations of population densities in the absence of farming, often describing imperial systems in Central Eurasia as unique due to their economic basis^65–69^. In other parts of Asia, farming is linked to demographic expansion and the congregation of greater population densities^70^. Notably, millet farming is linked to urbanization^71,72^ and imperial formation^73^ in East Asia. Boserupian economics suggest that increased investment in farming, along with a diversified economy and higher levels of cultural exchange, often lead to a demographic transition^74–76^. The data presented in this paper suggests that, while Mongolian empires have often been seen as outliers in global comparisons of imperial structures, they were in fact, like many others around the world, highly reliant on economic diversification, local adaptations to a diversity of environments, and the creation of reliable and stable subsistence resources and economic surpluses^8–10^.

Mongolian empires have traditionally conjured up exotic ideas of mobile pastoral specialists who roamed the Asian steppes attacking more sedentary communities^1–4^. While prominent in the public sphere, such preconceptions have also directed the type of questions academics have asked. For example, comparative analysis of Mongolian empires with others around the world has been limited, with ‘Steppe Empires’ often portrayed as deficient or somehow doomed to failure in the absence of reliable crop-based surplus^6,77^. As in other parts of Central Asia, where occupation sites have been hard to come by^78,79^, simplistic projections of ethnographic and ethnohistoric datasets into the past have been common in Mongolian archaeology. We hope to have demonstrated how multidisciplinary approaches, built on datasets from different parts of Mongolian imperial networks, can begin to provide novel insights into their economic systems and, perhaps most importantly, their geographic and temporal variability. While there is no doubt that the Xiongnu and Mongol empires were unique, they were also built upon many of the same tenants of economic diversity, stability, and reliability that have characterized imperial structures throughout prehistory and history, demonstrating the importance of a core set of underlying variables in both enabling and driving the formation of empires.

## Methods and Materials

### Sites and materials analyzed

All bone and tooth samples included in this study were collected from the National University of Mongolia’s Department of Archaeology during the winter of 2016. Bone collagen was analyzed for carbon (δ^13^C) and nitrogen (δ^15^N) stable isotopes, and the carbonate of dental enamel bioapatite was measured for carbon stable isotopes (δ^13^C), with some individuals analyzed for both bone collagen and bioapatite (see Supplementary Table 3). Time periods for samples ranged from the mid-fifth millennium B.C.E. to the Mongol Empire, as dated by AMS radiocarbon methods where possible (see below).

Samples of bones and teeth were collected from archaeological sites across the country of Mongolia, through varying environmental and topographical zones. Where possible, we collected a tooth and long bone fragment from individuals from each time period. While we aimed to assemble an equal number of samples from all time periods, the collection was dominated by individuals from the imperial periods, resulting in fewer individuals prior to the Iron Age. Bone collagen was preferably extracted from rib bones, but occasionally other bone fragments were employed (clavicle, femur, crania). δ^13^C and δ^15^N stable isotope measurements of human bone collagen inform primarily on protein source^80^, and the bones sampled (i.e. ribs) represent a period of diet of approximately the last 20 years of life^80^.

By contrast, tooth enamel  δ^13^C values are indicative of the whole dietary carbon (carbon mix of protein, lipids, and carbohydrates) consumed during enamel formation^33,49^. First molars mineralize before an individual is 3 years old, second molars are fully formed around age 8, and third molars, if present, are completely mineralized between the age of 7 and 16^81^. To avoid tooth samples that might show a breastfeeding isotopic signal contribution in older children and adolescents we preferentially selected second and third molars. First molars were chosen only when both the M2 and M3 were unavailable.

### Stable isotope analysis methods

#### Bone collagen

We selected ribs for bone collagen analysis as representative of the last *c*. 20 years of life^80^. Collagen was extracted from each rib sample following standard procedures^35^. Approximately 1 gram of pre-cleaned bone was demineralized in 10 ml aliquots of 0.5 M HCL at 4 °C, with changes of acid until CO_2_ stopped evolving. The residue was then rinsed three times in deionized water before being gelatinized in pH 3 HCl at 75 °C for 48 hours. The resulting solution was filtered, with the supernatant then being lyophilized over a period of 24 hours.

After calculating the collagen yield, all purified collagen samples (~1 mg) were located in tin capsules to be analyzed in duplicate at the Department of Archaeology, Max Planck Institute for the Science of Human History by the elemental analyzer/continuous flow isotope ratio mass spectrometry (EA-IRMS) using a ThermoFisher Elemental Analyzer coupled to a ThermoFisher Delta V Advantage Mass Spectrometer via a ConFloIV system. δ^13^C and δ^15^N values were compared and calibrated against International Standards (USGS40 (δ^13^Craw = −26.4 ± 0.1‰, δ^13^Ctrue = −26.4 ± 0.0‰, δ^15^Nraw = −4.4 ± 0.1‰, δ^15^Ntrue = −4.5 ± 0.2‰), IAEA N2 (δ^15^Nraw = +20.2 ± 0.1‰, δ^15^Ntrue = +20.3 ± 0.2‰), IAEA C6 (δ^13^Craw = −10.9 ± 0.1‰, ^13^Ctrue = −10.8 ± 0.0‰) Replicate analysis of an in-house fish gelatin standard suggests that machine measurement error is *c*. 0.1‰ for δ^13^C and 0.3‰ for δ^15^N.

#### Tooth enamel

Teeth or tooth fragments were cleaned using air-abrasion to remove any adhering external material. 8 mg of enamel powder was obtained using gentle abrasion with a diamond-tipped drill along the full length of the buccal surface or fragment in order to maximize the period of formation represented by the resulting isotopic analysis for bulk samples. Enamel powder was pre-treated using a protocol to remove any organic or secondary carbonate contaminates^21^. This consisted of a series of washes in 1.5% sodium hypochlorite for 60 minutes, followed by three rinses in purified H_2_O and centrifuging, before 0.1 M acetic acid was added for 10 minutes, followed by another three rinses in purified H_2_O (as per^35^).

Following reaction with 100% phosphoric acid, gases evolved from the samples were analyzed to stable carbon and oxygen isotopic composition using a Thermo Gas Bench II connected to a Thermo Delta V Advantage Mass Spectrometer at the Max Planck Institute for the Science of Human History, Jena (MPI-SHH). Carbon and oxygen isotope values were compared against an International Atomic Energy Agency (NBS 19) and in-house standard (MERCK). Replicate analysis of internal bovid enamel standards suggests that machine measurement error is *c*. ± 0.2‰ for δ^13^C and ± 0.2‰. Using a Thermo Gas Bench 2 in tandem with a Thermo Delta V Advantage Mass Spectrometer at MPI-SHH, gases produced from a reaction with 100% phosphoric acid were analyzed for stable carbon and oxygen isotopic composition. We compared the resulting values against International Standards (IAEA-603 (δ^13^C = 2.5; δ^18^O = −2.4); IAEA-CO-8 (δ^13^C = −5.8; δ^18^O = −22.7); USGS44 (δ^13^C = −42.2)); as well as an in-house standard of (MERCK (δ^13^C = −41.3; δ^18^O = −14.4)). The data from these standards suggest that the machine measurement error is *c*. ± 0.1‰ for δ^13^C and ± 0.2‰ for δ^18^O. We increased the precision of our analyzed samples by measuring repeats of extracts using a tooth enamel bovid standard (n = 20, ± 0.2‰ for δ^13^C and ± 0.3‰).

### Statistical tests

To determine whether the differences in human δ^13^C between each period were significant, we performed a Wilcoxon rank sum test, with multiple test correction using the Benjamini-Hochberg procedure. All tests were performed using the free R statistical software^82^.

### Bayesian dietary modelling

Caloric estimates of millet intakes were obtained using the Bayesian mixing model FRUITS having as input data individual tooth enamel δ^13^C values and local food isotopic values adjusted for spatial variations due to varying environmental conditions^80^. To achieve the latter, we grouped site locations into the categories of “steppe” and “dry” depending on modern day annual precipitation. Steppe sites have a range from 250–350 mm in precipitation per annum and arid sites have below 250 mm of yearly rainfall. It was assumed that the enamel δ^13^C signal is defined by the dietary carbon mix^50^. To extrapolate the spatial distribution of per capita millet caloric intakes (dietscape) a Bayesian additive mixed model with error-in variables^83–85^ available as an online app via the Pandora & IsoMemo initiatives was employed^86^. Dietscapes were generated for two main periods corresponding to a temporal divide defined by the intensification of millet consumption as observed from the interpretation of raw isotopic data, into Early (Bronze Age) and Late (combining the Early Iron Age; Xiongnu; Mongol periods). Modelling at a higher chronological resolution was not possible given a lack of data for shorter time periods. Further details on dietscape modelling are available in Supplementary Text S3.

### Radiocarbon and archaeologically classified dates

AMS radiocarbon dates were conducted at the Oxford Radiocarbon Accelerator Unit (ORAU), Oxford, England, UK (n = 14; bone collagen and dentine)^87^ and at the University of Groningen, Faculty of Science and Engineering, Groningen, The Netherlands (n = 25; bone collagen and dentine)^88^. All pre-Xiongnu samples and 30% of the Xiongnu samples were radiocarbon dated to solidify the dating of individuals from early time periods. Most Xiongnu and Mongol samples were separated into periods based on archaeological materials and burial styles as assessed by excavators and curators from the National University of Mongolia. See Supplementary Text S2 for additional details.

### Data availability and ethical approval statement

All of the data included in the study have been made available in Tables 1 and 2, the Supplementary Information. Samples analyzed for this study (AT- denoted codes) are currently curated at the Max Planck Institute for the Science of Human History, Jena, Germany. Samples were exported to the Max Planck Institute for the Science of Human History under permission from the Ministry of Culture, Education, Science and Sports (Export number 10/413 (7b/52) which was received on 2nd February, 2017 #A0109258, MN DE 7 643)

## Supplementary information

Supplementary Information.

Supplementary Table.

Supplementary Table S1.

Supplementary Table S2.

Supplementary Table S3.

Supplementary Table S4.
