# Supplementary material for: Economic Diversification Supported the Growth of Mongolia’s Nomadic Empires
Source: Sci Rep. 2020 Mar 3;10:3916. doi: 10.1038/s41598-020-60194-0 (PMC7054399; doi:10.1038/s41598-020-60194-0)
Supplement: Supplementary file 1 — Supplementary Information. [file 41598_2020_60194_MOESM1_ESM.pdf]

Supplementary Information

Economic Diversification Supported the Growth of Mongolia's Nomadic Empires

Wilkin, Shevan[1*], Alicia Ventresca Miller[1,2], Bryan K. Miller[1], Robert N. Spengler III[1], William T. T. Taylor[1,3], Ricardo Fernandes[1,4,5], Richard W. Hagan[6], Madeleine Bleasdale[1], Jana Zech[1], S. Ulziibayar[7], Erdene Myagmar[8], Nicole Boivin[1,9,10,11], Patrick Roberts[1]

1 Max Planck Institute for the Science of Human History, Department of Archaeology, Jena, Germany
2 University of Michigan, Department of Anthropology, Ann Arbor, Michigan, USA
3 University of Colorado, Department of Anthropology, Museum of Natural History, Boulder, CO, USA
4 School of Archaeology, University of Oxford, Oxford, UK
5 Faculty of Arts, Masaryk University, Brno, Czech Republic
6 Max Planck Institute for the Science of Human History, Department of Archaeogenetics, Jena, Germany
7 Institute of Archaeology and Ethnology, Mongolian Academy of Sciences, Jukoviin orgon chuloo 77, Ulaanbaatar, Mongolia
8 National University of Mongolia, Ulaanbaatar, Mongolia
9 School of Social Science, The University of Queensland, Brisbane, Australia
10 Department of Anthropology and Archaeology, University of Calgary, Calgary, Alberta, Canada
11 Department of Anthropology, National Museum of Natural History, Smithsonian Institution, Washington, D.C. USA

*correspondence to: Shevan Wilkin and Patrick Roberts
email: wilkin@shh.mpg.de, roberts@shh.mpg.de

This PDF file includes:

Supplementary Text 1: Stable carbon and nitrogen isotope analysis detailed background
Supplementary Text 2: AMS radiocarbon dating detailed methods
Supplementary Text 3: Bayesian dietary modelling and FRUITS methods
Supplementary Tables 3 to 7
References

Supplementary Text 1: Stable carbon and nitrogen isotope analysis detailed background for bone collagen and dental bioapatite

Stable carbon and nitrogen isotope ratios of human tissues are expressed in δ notation as ratios relative to established international standards and measured in parts per mil (‰). The delta values are obtained by the following equation: $[(R_{sample} - R_{standard)} / (R_{standard}) - 1] \times 1000$. Where the R represents the ratio between the heavier (^{13}C, ^{15}N) and lighter (^{12}C, ^{14}N) isotopes[1].

$\delta^{13}C$ variability in terrestrial ecosystems is primarily driven by two dominant photosynthetic pathways, C_3 and C_4, which differ in their net discrimination against ^{13}C during photosynthesis[2]. In C_3 plants, strong discrimination against ^{13}C during CO_2 fixation results in lower $\delta^{13}C$ values in virtually all trees, shrubs, and temperate grasses, including wheat and rice, than in C_4 plants such as millet (Farquhar et al, 1989). C_3 $\delta^{13}C$ values vary from *c.* -24 to -36‰ (global mean -26.5‰), while C_4 values range from *c.* -9 to -17‰ (global mean -12‰[2]). C_3 and C_4 plants therefore have distinct and non-overlapping $\delta^{13}C$ values as a product of their photosynthetic pathways[3]. These distinctions are reflected in the tissues of consumers of these plants[4]· The distribution of wild C_3 and C_4 plants in human ecosystems will, to some extent, be influenced by environmental conditions, with C_4 plants out-competing the former in warmer, more arid, and lower carbon dioxide conditions[5]· Even within the C_3 pathway, $\delta^{13}C$ can be affected by aridity, salinity, and temperature[6]. Finally, plants following an additional photosynthetic pathway, the Crassulacean Acid Metabolism pathway, can have $\delta^{13}C$ values within the C_3 to C_4 range, and often exist in arid regions[7].

These biological and ecological distinctions are passed into the tissues of consumers depending on the proportion of plants and animals (consumers of plants) consumed. Significantly, $\delta^{13}C$ analysis of human bone collagen primarily reflects the isotopic values of the protein input to the diet, with a minor contribution from lipids and carbohydrate[4]. This means that the $\delta^{13}C$ values of bone collagen will be heavily influenced by protein-rich foods[4,8]. For bone collagen, $\delta^{13}C$ values for 100% C_3 protein and energy diets, in open settings, average around –22‰ and those of 100% C_4 would be –8‰[9]. In contrast to bone collagen, $\delta^{13}C$ measurements of tooth enamel bioapatite reflect the carbon in the 'whole-diet' during the period of enamel formation that will vary depending on species and tooth sampled[10]. In the pre-fossil fuel era, average values for herbivores feeding in an open C_3 and C_4 landscape would be about -12‰ and 0‰, respectively[11,12], though different diet-enamel spacing for humans could lead to some variability in this regard[9,13]. Significantly, however, these baselines will vary with external environmental conditions. This makes it important to build detailed, contextual understandings of natural availability of C_3 and C_4 plants, as well as climatically driven $\delta^{13}C$ variation, on the bases of associated fauna or plants samples if isotopic differences are to be interpreted as real cultural choices.

In bone collagen, stable nitrogen isotope ratios ($\delta^{15}N$) provide additional dietary insights. Animal $\delta^{15}N$ varies with trophic level, and $\delta^{15}N$ trophic shifts of +2.7 to +3.3‰ per level are well documented in marine and terrestrial systems[8]. This trophic effect is seemingly a result of the loss of ^{15}N-depleted products during excretion, though it should be noted that diet-tissue distinctions are highly variable between animals[14]. Freshwater fish typically have higher $\delta^{15}N$ values than terrestrial fauna although $\delta^{13}C$ values of terrestrial and freshwater sources often overlap[8]. The $\delta^{15}N$ of plants, as well as their consumers, also has the potential to be influenced by environmental conditions, including aridity, salinity, and soil fertility[15-17]. In the context of human managed landscapes, manuring can also lead to higher $\delta^{15}N$ values in plants and consumers of those plants[18]

Supplementary Text 2: AMS radiocarbon dating detailed methods

Groningen: Samples were decalcified over at least a 24-hour period using mild acid (HCl, 2-4% w/vol; RT) at the Center for Stable Isotope Research at the University of Groningen. For each sample still not fully decalcified, we refreshed the solution, removing and storing soft portions separately in demineralised water until further preparation. Soft and pliable fragments were rinsed thoroughly with demineralised water. Extracts were then exposed to NaOH (1%, ~30 min) to eliminate humic acids, rinsed to neutrality and treated once more with acid (HCl, 4% w/vol, 15 min). The raw collagen fraction was denatured to gelatin in acidified demineralised water (pH 3) at 80 °C for 18 hours. Before drying, the dissolved gelatin was filtered through a 50 µm mesh to eliminate any remaining foreign particulates, and the crystalline collagen scraped from the glass. Approximately 4 mg aliquots of the reduced carbon fraction were then weighed into tin capsules for combustion in an Elemental Analyser (EA, IsotopeCube NCS, Elementar®). The EA was coupled to an Isotope Ratio Mass Spectrometer (IRMS, Isoprime® 100), allowing the $\delta^{13}C$ value of the sample to be measured, as well as a fully automated cryogenic system to trap CO_2 liberated on combustion. After run completion, the individual reaction vessels were transferred to a graphitisation manifold, where a stoichiometric excess of H2 gas (1: 2.5) was added, and the CO_2 gas reduced to graphite over an Fe(s) catalyst. The graphite samples were then pressed, and the radioisotopic ratio determined on a MICADAS accelerator mass spectrometer.

Oxford: The ORAU followed routine pre-treatment and measurement procedures[23]. For each specimen, between 200-600mg of bone or dentine was drilled using a handheld dentist drill, and collagen was extracted through a series of chemical steps that involved immersion in HCl, removal of humic acids using NaOH and removal of adsorbed CO_2 via a final HCl wash. Only 4 of the samples prepared at the ORAU (OxA-36230, -36231, -36232, -36233; indicated with an asterisk) underwent ultrafiltration using Vivaspin ultrafilters, due to initial indications of poor collagen perseveration. Extracted collagen was frozen overnight and was lyophilized. Between 2-5 mg of collagen was combusted in an elemental analyser (EA) and its C and N stable isotopes were measured at an IRMS instrument linked to the EA, before excess gas CO2 was collected, graphitized and measured at an HVEE accelerator, alongside blanks and standards. These were used for contamination calculation and final correction of the data. Collagen yields ranged greatly from 0.8% to 17.8%, and the C:N ratio of the extracted collagen fell within expected ranges (3.2-3.4) with the exception of OxA-36233 (C:N=3.6) and %C in the combusted collagen was between 37-46%.

Supplementary Text 3: Bayesian dietary modelling

The local isotopic baseline for each archaeological site was defined according to two main environmental classifications: dry and steppe. The $\delta^{13}C$ values for past C_3 plants in each environment was estimated from modern references using data available from Stacy 2008 (δ^{13}Cdry = -26.9±1.2‰, n = 9; δ^{13}Csteppe = -25.3±1.4‰, n = 20). To these values a correction (+1.85‰) was applied to account for the Suess effect, the variation in the δ13C value of atmospheric CO2 between the collection period and the archaeological period under study. The reference $\delta^{13}C$ value for millet grains (δ^{13}Cdry = -9.9±0.6‰) was taken as the weighted average of reported means for archaeological charred millet grains in the western part of the Chinese Loess Plateau[24].

The $\delta^{13}C$ values of archaeological bone collagen from domesticated herbivores from six sites were employed as reference for dry and steppe sites ($\delta^{13}C$ dry = -16.5±1.3‰, n = 11; $\delta^{13}C$ steppe = -19.3±1.3‰, n = 34). The $\delta^{13}C$ values of lipids and protein in terrestrial animal products (e.g. meat, milk) were estimated following the same procedure as described in Fernandes et al[25] by adding offsets to bone collagen values of -8‰ and -2‰, respectively. The macronutrient caloric composition of terrestrial animal products was set at 70±10% lipids and 30±10% carbohydrates as described in Fernandes et al [25]. The bulk composition of terrestrial animal products was estimated from a weighted average of macronutrient isotopic and caloric compositions.

The Bayesian software FRUITS 3.0 was employed to reconstruct, from tooth enamel $\delta^{13}C$ measurements, millet caloric contributions towards individual diets[26]. It was assumed that the carbon in tooth enamel was sourced from the scrambled dietary carbon pool[13]. An isotopic offset of -12‰ between diet and tooth enamel was taken as reference[27]. The models employed for dietary estimates are fully described as supplementary FRUITS files (Dry.frt; Steppe.frt).

To extrapolate the spatial distribution of millet consumption in Mongolia during two main periods, Early (4400 – 800 B.C.E.) and Late (c. 800 B.C.E. – 1400 C.E.), a Bayesian additive mixed model with error-in variable was employed[28,29] by assuming that the estimates for millet consumption follow a Gaussian distribution. The spatial mean of millet estimates were defined by a 2-dimensional smooth function depending on latitude and longitude using the model AverageR available through the online app developed within the Pandora & IsoMemo initiatives[30].

Supplementary Table 5 The results (p – values) of a Wilcoxon rank sum test with correction via Benjamini-Hochberg to determine whether the differences in δ13C is greater between periods than within them.

Group	Early	Early Iron	Mongol	Faunal
Early Iron	0.0129	-	-	-
Xiongnu	0.0029	0.9795	0.1286	4.4e-09
Mongol	0.0517	0.2531	-	9.8e-06
Faunal	0.0311	0.0017	-	-

Supplementary Table 6: The results (p – values) of a Wilcoxon rank sum test with correction via Benjamini-Hochberg to determine whether the differences in δ13C is greater between periods than within them.

Group	Early	Early Iron	Mongol
Early Iron	0.00028	-	-
Xiongnu	9.1e-05	0.916	0.207
Mongol	1.4e-05	0.393	-

Supplementary Table S7: *The results (p – values) of a Wilcoxon rank sum test with correction via Benjamini-Hochberg to determine whether the differences in δ15N is greater between periods than within them.*

Group	Early	Early Iron	Mongol
Early Iron	0.075	-	-
Xiongnu	0.020	0.953	0.953
Mongol	0.075	.953	-

References

1. Roberts, P. *et al.* Fruits of the forest: Human stable isotope ecology and rainforest adaptations in Late Pleistocene and Holocene (~36 to 3 ka) Sri Lanka. *Journal of Human Evolution* **106**, 102–118 (2017).
2. Smith, B. N. & Epstein, S. Two Categories of 13C/12C Ratios for Higher Plants. *Plant Physiology* **47**, 380–384 (1971).
3. Tieszen, L. L. Natural variations in the carbon isotope values of plants: Implications for archaeology, ecology, and paleoecology. *J. Archaeol. Sci.* **18**, 227–248 (1991).
4. Ambrose, S. H. & Norr, L. Experimental Evidence for the Relationship of the Carbon Isotope Ratios of Whole Diet and Dietary Protein to Those of Bone Collagen and Carbonate. *Prehistoric Human Bone* 1–37 (1993). doi:10.1007/978-3-662-02894-0_1
5. Farquhar, G. D., Ehleringer, J. R. & Hubick, K. T. Carbon Isotope Discrimination and Photosynthesis. *Annu. Rev. Plant Physiol. Plant Mol. Biol.* **40**, 503–537 (1989).
6. Heaton, T. H. E. Spatial, Species, and Temporal Variations in the13C/12C Ratios of C3Plants: Implications for Palaeodiet Studies. *J. Archaeol. Sci.* **26**, 637–649 (1999).
7. O'Leary, M. H. Carbon isotopes in photosynthesis. *Bioscience* **38**, 328–336 (1988).
8. Schoeninger, M. J. & DeNiro, M. J. Nitrogen and carbon isotopic composition of bone collagen from marine and terrestrial animals. *Geochim. Cosmochim. Acta* **48**, 625–639 (1984).
9. Kellner, C. M. & Schoeninger, M. J. A simple carbon isotope model for reconstructing prehistoric human diet. *Am. J. Phys. Anthropol.* **133**, 1112–1127 (2007).
10. Passey, B. H. *et al.* Carbon isotope fractionation between diet, breath CO2, and bioapatite in different mammals. *J. Archaeol. Sci.* **32**, 1459–1470 (2005).
11. Lee-Thorp, J. A., Sealy, J. C. & van der Merwe, N. J. Stable carbon isotope ratio differences between bone collagen and bone apatite, and their relationship to diet. *J. Archaeol. Sci.* **16**, 585–599 (1989).
12. Levin, N. E., Simpson, S. W., Quade, J., Cerling, T. E. & Frost, S. R. Herbivore enamel carbon isotopic composition and the environmental context of Ardipithecus at Gona, Ethiopia. *Geol Soc Am Spec Pap* **446**, 215–234 (2008).
13. Fernandes, R., Nadeau, M.-J. & Grootes, P. M. Macronutrient-based model for dietary carbon routing in bone collagen and bioapatite. *Archaeol. Anthropol. Sci.* **4**, 291–301 (2012).
14. Hedges, R. E. M. & Reynard, L. M. Nitrogen isotopes and the trophic level of humans in archaeology. *J. Archaeol. Sci.* **34**, 1240–1251 (2007).
15. Amundson, R. *et al.* Global patterns of the isotopic composition of soil and plant nitrogen. *Global Biogeochem. Cycles* **17**, (2003).
16. Ometto, J. P. H. B. *et al.* The stable carbon and nitrogen isotopic composition of vegetation in tropical forests of the Amazon Basin, Brazil. *Biogeochemistry* **79**, 251–274 (2006).
17. Craine, J. M. *et al.* Global patterns of foliar nitrogen isotopes and their relationships with climate, mycorrhizal fungi, foliar nutrient concentrations, and nitrogen availability. *New Phytol.* **183**, 980–992 (2009).
18. Bogaard, A., Heaton, T. H. E., Poulton, P. & Merbach, I. The impact of manuring on nitrogen isotope ratios in cereals: archaeological implications for reconstruction of diet and crop management practices. *J. Archaeol. Sci.* **34**, 335–343 (2007).
19. Hedges, R. E. M., Clement, J. G., Thomas, C. D. L. & O'Connell, T. C. Collagen turnover in the adult femoral mid-shaft: Modeled from anthropogenic radiocarbon tracer measurements. *American Journal of Physical Anthropology: The Official Publication of the American Association of Physical Anthropologists* **133**, 808–816 (2007).

20. Richards, M. P. & Hedges, R. E. M. Stable Isotope Evidence for Similarities in the Types of Marine Foods Used by Late Mesolithic Humans at Sites Along the Atlantic Coast of Europe. *J. Archaeol. Sci.* **26**, 717–722 (1999).
21. Lee-Thorp, J. *et al.* Isotopic evidence for an early shift to C4 resources by Pliocene hominins in Chad. *Proc. Natl. Acad. Sci. U. S. A.* **109**, 20369–20372 (2012).
22. Roberts, P. *et al.* Direct evidence for human reliance on rainforest resources in late Pleistocene Sri Lanka. *Science* **347**, 1246–1249 (2015).
23. Brock, F., Higham, T., Ditchfield, P. & Ramsey, C. B. Current Pretreatment Methods for AMS Radiocarbon Dating at the Oxford Radiocarbon Accelerator Unit (Orau). *Radiocarbon* **52**, 103–112 (2010).
24. An, C.-B. *et al.* Stable isotopic investigations of modern and charred foxtail millet and the implications for environmental archaeological reconstruction in the western Chinese Loess Plateau. *Quaternary Research* **84**, 144–149 (2015).
25. Fernandes, R., Grootes, P., Nadeau, M.-J. & Nehlich, O. Quantitative diet reconstruction of a Neolithic population using a Bayesian mixing model (FRUITS): The case study of Ostorf (Germany). *Am. J. Phys. Anthropol.* **158**, 325–340 (2015).
26. Fernandes, R., Millard, A. R., Brabec, M., Nadeau, M.-J. & Grootes, P. Food reconstruction using isotopic transferred signals (FRUITS): a Bayesian model for diet reconstruction. *PLoS One* **9**, e87436 (2014).
27. Tykot, R. H. Stable isotopes and diet: you are what you eat. in *Proceedings-International School of Physics Enrico Fermi* **154**, 433–444 (IOS Press; Ohmsha; 1999, 2004).
28. Groß, M. Modeling body height in prehistory using a spatio-temporal Bayesian errors-in variables model. *AStA Adv. Stat. Anal.* **100**, 289–311 (2016).
29. Rosenstock, E., Ebert, J., Martin, R., Hicketier, A., Walter, P., Groß, M. Human Stature in the Near East and Europe ca. 10 000 – 1000 BC: its spatio-temporal development in a Bayesian errors-in-variables model. *Archaeol. Anthropol. Sci.*
30. Fernandes, R., Salesse, K., Larsen, T., Knipper, C., Feng, F., Wang, Y. IsoMemo: a Big isotopic Data initiative for archaeology, ecology, and environmental & life sciences. *IsoMemo* Available at: http://www.isomemo.com. (Accessed: 27th May 2019)
